# An Unexpected Finding of a Papillary Fibroelastoma in the Left Ventricle of an Asymptomatic Patient—A Case Report

**DOI:** 10.3390/reports8020090

**Published:** 2025-06-06

**Authors:** Nicole Piber, Christian Nöbauer, Bernhard Voss, Markus Krane, Stephanie Voss

**Affiliations:** Department of Cardiovascular Surgery, German Heart Center Munich, School of Medicine & Health, Technical University of Munich, Institute Insure, Lazarettstraße 36, 80636 Munich, Germany

**Keywords:** papillary fibroelastoma, left ventricular mass, surgical techniques, case report

## Abstract

**Background and Clinical Significance:** Papillary Fibroelastoma is a benign primary cardiac tumor, commonly located in a valvular position, predominantly on the aortic valve. **Case Presentation**: We present a 73-year-old male patient with a medical history of chronic lymphatic leukemia, kidney failure, diabetes, and obstructive sleep apnea. In a routinely performed echocardiogram an abnormal structure in the left ventricle was found. The patient presented completely asymptomatically at the time of examination. A cardiac magnetic resonance-scan provided further information about the size and localization of the tumor in the left ventricle, which seemed to be attached to a papillary muscle and was about 1.6 cm in diameter. Due to visible scarring of the myocardia, which was identified in the scan, a cardiac catheter examination was performed. A coronary artery disease was detected with a severe stenosis in three vessels. During an elective bypass-operation, the removal of the structure was performed with an approach through the left atrium, passing the mitral valve using a valve sizer for better exposure. The tumor of 1 cm presented macroscopically with an anemone-like shape. The histopathological examination confirmed the intraoperative assumption of a papillary fibroelastoma, found in an aberrant location. **Conclusions**: Unexpectedly challenging surgical removals of structures in the left ventricle require innovative techniques with available instruments for better exposure.

## 1. Introduction and Clinical Significance

Primary cardiac tumors are rare and mainly benign with an incidence of 0.056% [1]. The cardiac myxoma is the most common cardiac tumor, present in about 76,5% of all primary cardiac tumors, followed by the papillary fibroelastoma (PFE) in 12.7% [2]. Uncertainties have evolved around the origin of the PFE. The prevalent theory is that the tumor emerges from microthombotic lesions rather than preexisting congenitally [3]. The finding of fibrin and laminated elastic fibers, which are also found in thrombus formations, support this theory [4].

Due to impaired blood flow around the structure, hemostasis occurs and clots form. The detachment of clots provokes embolism, the most common clinical symptom of PFEs [5]. Heterogeneous neurological symptoms are described in the literature, ranging from hemiparesis and aphasia to visual impairment [6,7,8]. After the onset of neurological symptoms, cardiac masses are detected through echocardiographic examination to identify cardioembolic sources. The second most common symptom is heart failure followed by sudden cardiac death. Myocardial infarction or death occur due to a prolapse of the tumor into the valve orifice or coronary ostia occlusion [9,10]. About one third of patients show no symptoms at diagnosis [11].

## 2. Case Presentation

An asymptomatic 73-year-old male patient presented for a routine cardiac examination due to chronic lymphocytic leukemia (CLL) for the first time. Aside from CLL, his medical history showed kidney failure, diabetes type II, obesity, and obstructive sleep apnea. No cardiac predisposition was known. He had no history of thrombosis or thrombophilia. There were no reported symptoms of dyspnea, chest pain, or vertigo. The patient was classified as NYHA (New York Heart Association) class I, indicating no limitation of physical activity. At the time of presentation, there were no neurological symptoms suggestive of stroke or embolism.

### 2.1. Diagnostic Assessment

**Figure 1:**
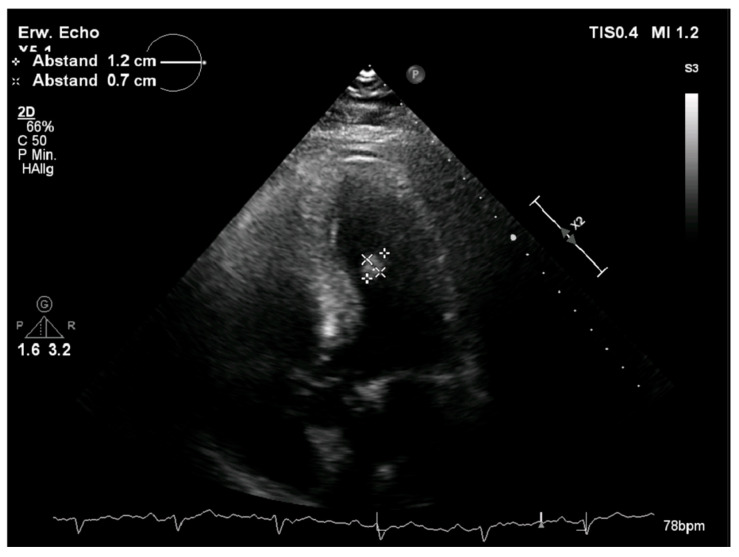
Preoperative echocardiographic examination of the left ventricle. The tumor is measured and marked with white crosses.

**Figure 2:**
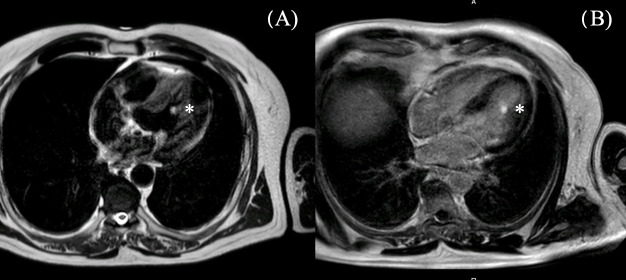
CMR examination of the tumor preoperatively. The tumor is marked with * (**A**) T2W_TSE sequence (**B**) 3D LE 4CH with late enhancement of the tumor.

Through the routinely performed transthoracic echocardiography, a structure in the left ventricle was detected. It appeared round, smooth-surfaced with a diameter of 1.2 × 0.7 cm, and located on the lateral wall of the left ventricle (Figure 1). The intracardiac mass appeared to be oscillating in the left ventricle without causing obstructions or valve insufficiency. The left ventricle showed a normal ejection fraction of 58% with no motion abnormalities. There was no valvular pathology. For further investigation of the tumor, a cardiac magnetic resonance (CMR) with a gadolinium contrast agent was performed. The CMR showed a smooth-surfaced structure on T2-weighted images with a diameter of 12 mm in adhesion to the papillary muscle with homogeneous signal intensity and late gadolinium enhancement (Figure 2). Additionally, the CMR showed visible scarring of the myocardium in various locations. To investigate the probability of coronary artery disease causing the scarring, a cardiac catheter examination was performed. In the catheter examination, a coronary heart disease of three vessels was detected. The left anterior descending artery showed a severe long-distance stenosis of 90%, the left circumflex artery was affected with a stenosis of 60%, and the intermediate artery presented with a severe stenosis of 90%. Due to these findings, the indication for a coronary bypass operation was met. The surgical procedure was planned accordingly.

### 2.2. Therapeutic Intervention

The patient was referred to our department of cardiovascular surgery for the urgent removal of the ventricular tumor and simultaneous coronary bypass treatment. Intracardiac masses are a source of emboli that can cause severe damages, such as strokes or pulmonary embolisms. Therefore, a prompt removal is mandatory. After completing preoperative examinations, the patient was transferred to surgery. The preoperative ECG showed a left anterior hemiblock. As the initial cardiac examination was the first, no prior ECGs were available.

Intraoperative transesophageal echocardiography showed a good ejection fraction and a mild mitral regurgitation. The presence of a structure in the left ventricle, measuring 1.1 × 1.3 cm, was confirmed. The tumor originated from the antero-lateral papillary muscle at the offspring of the leaflet chordae. It appeared to be oscillating into the cavity of the left ventricle. Additionally, a persistent foramen ovale (PFO) was detected.

**Figure 3:**
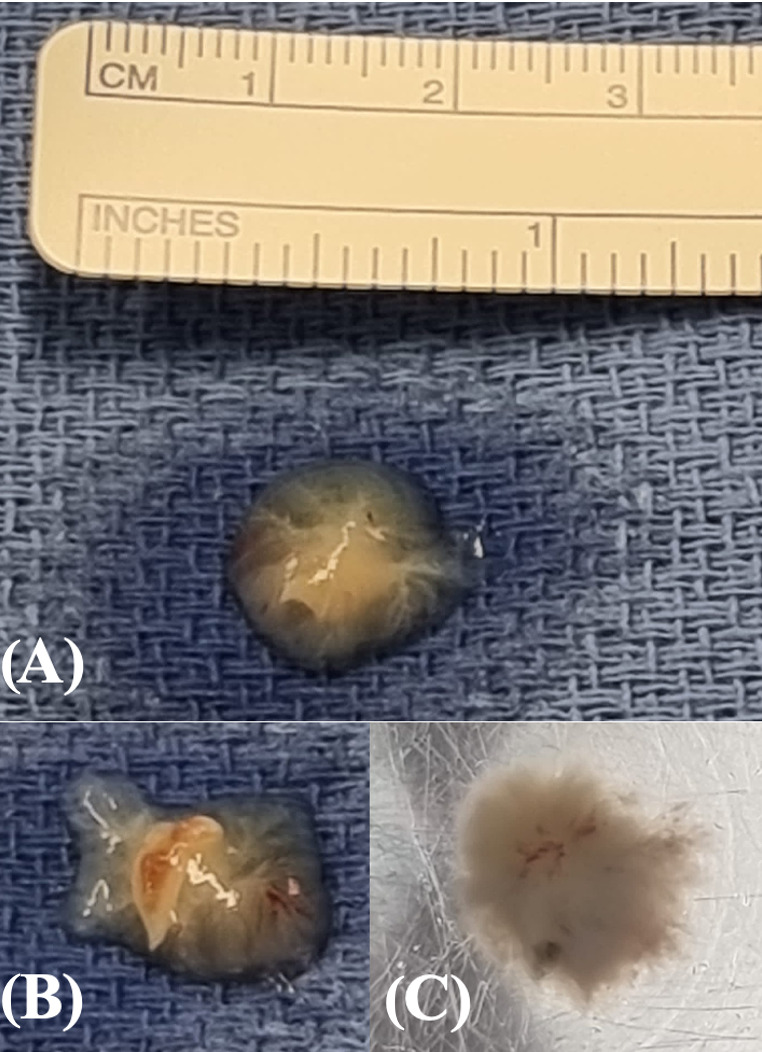
Fibroelastoma after removal: (**A**) view from above, (**B**) visible cut surface (**C**) in fluid.

After performing a median sternotomy, the bypass material was harvested. The preparation of the left internal mammary artery (LIMA) was performed simultaneously alongside the endoscopic harvesting of the right saphenous vein graft. A cardio-pulmonary bypass was established through a cannulation of the aorta and venae cavea. Mild hypothermia of 32 °C was achieved. The heart was not manipulated before aortic clamping to avoid any detachment of the tumor and possible embolization. After the clamping of the aorta, cardiac arrest was obtained via the instillation of 1500 mL of Bretschneider’s cardioplegic solution. After cardiac arrest, the coronary arteries were inspected. The right coronary artery was unsuitable for bypass treatment due to severe calcification. Distal anastomosis of the intermediate artery with the veinous graft and LIMA end-to-side anastomosis with the left anterior descending artery for coronary revascularization were performed. Next, the left atrium was accessed and the PFO was closed through a direct suture. The exposure of the tumor was challenging due to its anatomical location. Initially, stay sutures were added to the annulus of the mitral valve for better exposure. The tumor was still not accessible through the mitral valve. As described in a previous report, we use aortic valve seizers for mitral valve repair with neochordae for better visualization of the chordae at our institution [12]. This technique minimizes leaflet manipulation. As the tumor was located at the offspring of the chordae from the papillary muscle, a position similar to the insertion of the neochordae in mitral valve repair, an analogous approach was implemented. A commercially available 27 mm valve sizer for aortic valve prostheses (Trifecta, St. Jude Medical, Minneapolis, MN, USA) was inserted into the mitral valve, repressing the leaflets without force, displacement, or damage. With an optimized visualization of the chordae, the tumor was exposed properly and could be removed in toto (Video S1). It presented macroscopically in an anemone-like shape, which is characteristic for papillary fibroelastomas, with a diameter of 1 cm (Figure 3). The seizer and the stay sutures were removed carefully from the mitral valve. Afterwards, the mitral valve was tested and appeared competent. The left atrium was closed. The patient was referred to the intensive care unit. No neurological symptoms were apparent during the intrahospital course. The postoperatively performed transthoracic echocardiogram showed satisfactory results with sufficient left ventricular ejection fraction. No residues of the intracardiac tumor could be detected. The patient was discharged on day eight post-operation. No major complications occurred during follow-up.

### 2.3. Outcomes

**Figure 4:**
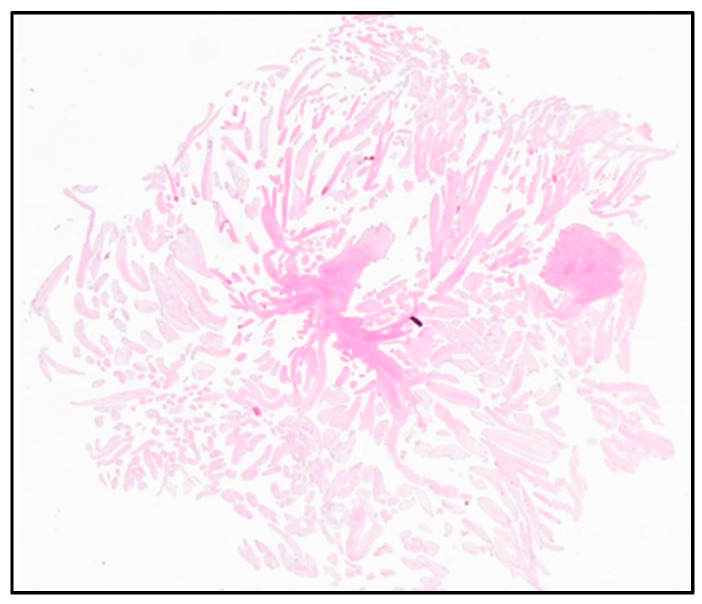
Histological examination: small avascular papillary fronts with branchlike ramifications, merging into a central stalk consisting of fibroelastic fibers.

The resected tumor measured 1 × 1 × 0.6 cm and appeared to be soft and white. In the histopathological examination, small avascular papillary fronts with branchlike ramifications were detected merging into a central stalk consisting of fibroelastic fibers. The tumor was covered in bland endothelium (Figure 4). The histological examination resulted in the diagnosis of a PFE.

## 3. Discussion

In this case, the PFE was located in the left ventricle, attached to a papillary muscle. In previous studies, the most common locations for PFE have been the aortic valve in 44% of cases, followed by the mitral and the tricuspid valve in 35% and 15% of cases, respectively. PFEs located on the ventricular side are rare and account for only 10% of all PFEs [13]. Only a few cases of left ventricular PFEs have been reported so far [14].

Clinically, most patients present as asymptomatic. About 30% of all papillary fibroelastomas are detected due to neurological symptoms, requiring echocardiography to exclude a cardioembolic source [15]. Cardiac masses are rare but must be considered a significant differential diagnosis in patients presenting with a stroke and no prior risk factors or medical history.

The surgical removal of left ventricular structures is not a standardized procedure. Steps must be adapted to the individual anatomy and location of the tumor. The favored access leads through the left atrium, passing the mitral valve [16]. Due to abnormalities in the anatomy of the mitral valve—for example, excessive leaflet tissue—the exposure of the tumor can be challenging. In our case, we used a valve seizer for better exposure and to protect the leaflets during removal. This method is used routinely for mitral valve repair with neochordae at our institution [12]. Another approach for PFEs in the left ventricle is the transapical approach through left apical ventriculotomy [17]. This method is used in cases where the tumor is located at the apex of the heart or the approach through the mitral valve is not possible because of earlier mechanical valve implantation.

In rare cases, patients decide against surgical removal and have to receive anticoagulants. Yiu et. Al. describe the case of a 58-year old female diagnosed with a PFE, deciding on non-surgical treatment, who remained asymptomatic on low dose aspirin [18].

For future encounters with ventricular masses, one must be aware of unexpected challenges concerning the exposure of the tumor. Standard utensils for cardiac operations can be used to optimize accessibility. The use of a valve sizer is an atraumatic way to repress the mitral leaflets and shield them at the same time from unintentional damage during the retrieval of the tumor from the left cavity.

## 4. Conclusions

In conclusion, the removal of a papillary fibroelastoma is a safe procedure which can bear unexpected challenges that are in need of the innovative use of preexisting instruments for better exposure.

## Data Availability

The original contributions presented in this study are included in the article. Further inquiries can be directed to the corresponding author.

## Supplementary Materials

The following supporting information can be downloaded at: https://www.mdpi.com/article/10.3390/reports8020090/s1, Video S1: Video of intraoperative transesophageal echocardiography, retrieval of the tumor, and histo-pathological examination: First sequence: position of the tumor in the left ventricle and tumor measurements. Second sequence: intraoperative view exposing the tumor using a valve seizer. Third sequence: excised tumor followed by histological image of the tumor.

## Abbreviations

PFE	papillary fibroelastoma
CLL	chronic lymphatic leukemia
NYHA	New York Heart Association
CMR	cardiac magnetic resonance
LIMA	left internal mammary artery
PFO	persistent foramen ovale
